# New onset diabetes manifesting as diabetic ketoacidosis in a patient with chronic myelogenous leukemia treated with imatinib

**DOI:** 10.20945/2359-4292-2024-0432

**Published:** 2025-04-03

**Authors:** Dinara Salimova, Tatevik Aloyan, Ursula Qureshi, Faisal Qureshi, Hina Wazir

**Affiliations:** 1 Ascension St Joseph Hospital, Chicago, IL, USA; 2 Kansas City University College of Medicine and Biomedical Sciences, Kansas City, MO, USA

## Abstract

Imatinibis a commonly used antiproliferative agent for treating chronic myelogenous leukemia and gastrointestinal stromal tumors, and it is also thought to be effective in other areas, such as rheumatologic diseases. It has been shown to improve glucose control in diabetic patients by lowering blood sugar, reducing HbA1c levels, and decreasing the need for diabetes medications. However, we present a rare occurrence of severe hyperglycemia and newly elevated HbA1c in a patient on imatinib with no prior history of diabetes. It underscores the need for further research to assess the safety and impact of imatinib on glucose metabolism.

## INTRODUCTION

Imatinib is a targeted tyrosine kinase receptor inhibitor used to treat Philadelphia chromosome-positive chronic myelogenous leukemia and gastrointestinal stromal tumors. Its introduction marked a significant breakthrough in cancer treatment, being the first anti-cancer drug specifically designed to target a molecular abnormality found in cancer cells (^1^). Imatinib has shown a positive impact on diabetes patients, with multiple case reports and trials indicating its ability to lower blood glucose levels, HbA1c, and reduce insulin needs in both type 1 and type 2 diabetes (^2-5^). Interestingly, we encountered a case of newly diagnosed diabetes, presenting with severe hyperglycemia and diabetic ketoacidosis, in a patient who had been on imatinib therapy. To our knowledge, this is the first reported instance of new-onset diabetes in a patient undergoing imatinib treatment. This case underscores the need for further research to evaluate imatinib’s safety and efficacy in relation to glucose metabolism.

## CASE REPORT

A 63-year-old male of Indian descent with a medical history of hypertension, stage 3a chronic kidney disease, hepatosteatosis, and chronic myelogenous leukemia (CML) managed with imatinib presented to follow up oncology appointment with a blood glucose of 822 mg/dL. He was advised to go to the emergency department and was subsequently admitted to the Internal Medicine floor on the same day for treatment of diabetic ketoacidosis.

The patient has a longstanding history of chronic phase CML since 2012, which has been treated with imatinib for at least a year before his presentation. Bone marrow biopsy results after initiation of imatinib indicated a major molecular response to treatment. At the time of admission, the patient’s medication regimen included imatinib mesylate 400 mg daily, amlodipine 10 mg daily, losartan 100 mg daily, and metoprolol 50 mg twice daily. He reported symptoms of polyuria, nocturnal bilateral foot spasms and noted a weight loss of 25 pounds over the past 3 months. The patient denied experiencing symptoms such as burning during urination, cough, shortness of breath, fever, chills, diarrhea, joint pain or swelling, skin lesions, or other signs suggestive of infection. Additionally, he reported no family history of diabetes.

Upon presentation, vital signs indicated that the patient was hemodynamically stable with blood pressure ranging from 130s to 150s mmHg systolic over 70-80s mmHg diastolic with a normal respiratory rate and oxygen saturation on room air. The patient was afebrile, with a normal heart rate. His BMI of 38.4 was notable for class II obesity. Physical examination was largely unremarkable and the patient was alert and fully oriented.

The complete blood count was within normal limits, with no abnormality in white blood cell count, hemoglobin level, or platelet count. Urinalysis did not show evidence of infection, and chest x-ray was unremarkable. The significant results from laboratory workup are outlined in Table 1.

**Table 1:** Laboratory analyses

	Values	Units	Reference values
Serum glucose	456	mg/dL	7-99
Serum creatinine	1.44	mg/dL	0.7-1.3
GFR	55	mL/min/1.73 m^2^	>60
Blood ketones, POC	2.7	mmol/L	<0.6
Potassium	4.1	mmol/L	3.5-5.2
Chloride	95	mmol/L	98-107
Sodium	131	mmol/L	133-144
Bicarbonate	20	mmol/L	21-31
Anion gap	16	mmol/L	6-15
HbA1c	12.9	%	<5.7
AST	47	IU/L	13-39
ALT	78	IU/L	7-52
Urine ketones	20	mg/dL	negative
Urine glucose	>500	mg/dL	negative
Urine protein	100	mg/dL	negative

The patient was diagnosed with diabetic ketoacidosis. He was initiated on insulin therapy, which led to the resolution of the anion gap metabolic acidosis and hyperglycemia.

Upon discharge, he was prescribed insulin glargine 20 units daily and Humalog 7 units with meals. Imatinib was discontinued, and he was started on another regimen for treatment of CML.

The patient returned for follow-up visit with an endocrinologist 3 months after his initial presentation, where testing revealed a C-peptide level of 0.4 ng/mL and negative results for autoantibodies, including those against glutamate decarboxylase, islet antigen-2, insulin, and zinc transporter-8. His HbA1c had decreased to 11.5%. Insulin therapy was maintained, and the patient was advised to continue follow-up at our clinic.

## DISCUSSION

Imatinib was the first tyrosine kinase inhibitor approved by FDA for treating Philadelphia chromosome-positive CML (^6^). Its effectiveness has significantly improved patient prognosis by targeting the Abelson (ABL) tyrosine kinase, which is aberrantly expressed as a deregulated protein BCR-ABL (^7,8^). Additionally, imatinib has been found to potently antagonize several other protein kinases, including c-KIT, PDGF receptors, c-Fms, c-Abl and potentially Lck. This broad kinase inhibition makes it effective in the treatment of other cancers, such as gastrointestinal stromal tumors (^3,4^). Consequently, imatinib is now widely utilized in oncology for the treatment of a variety of malignancies.

Literature indicates that imatinib tends to lower free plasma glucose levels and HbA1c concentrations in patients with both type 1 and type 2 diabetes (^4,9,10^). Lavelle and cols. reported a case of type 1B diabetes in an adolescent where partial diabetes remission was maintained for at least 6 months following initiation of imatinib therapy (^4^). Similarly, Salaroli and cols. observed a reduction in fasting serum glucose, HbA1c, and insulin requirements in a 24-year-old patient with chronic myeloproliferative disease receiving imatinib (^10^). Additionally, several cases have documented decreased fasting glucose level and HbA1c level in patients with type 2 diabetes. For example, Veneri and cols. described a case of a 70-year-old woman with type 2 diabetes who was able to discontinue insulin treatment after 4 months of imatinib therapy for CML (^3^).

The exact mechanism by which imatinib beneficially alters glucose metabolism remains unclear, but it is thought that it may be linked to the drug’s inhibition of key proteins involved in the insulin signaling pathway (^9^). For instance, imatinib inhibits the non-receptor tyrosine kinase c-Abl, which plays a significant role in various signaling pathways. C-Abl is known to become highly activated in the setting of cellular stress or damage, leading to cell cycle arrest and apoptosis. In mouse models of both type 1 and type 2 diabetes, imatinib has been shown to prevent beta-cell death and destruction by inhibiting c-Abl (^11^). Another potential mechanism involves the downregulation of PDGFRA and PDGFRB, which participate in adipogenesis and adiponectin secretion (^12^). Additionally, imatinib’s inhibition of EGFR has been associated with improved insulin sensitivity through reduced expression of TNF-alpha and IL-6 resulting in decreased infiltration of M1 macrophages in adipose tissue (^13^).

While there are promising findings suggesting imatinib’s positive effects on glucose metabolism in both pre-clinical and clinical studies, our case presents a notable exception: a patient with the onset of hyperglycemia and diabetes although he underwent treatment with imatinib for CML for at least a year.

In our case, a 63-year-old male of Indian descent, with no family history of diabetes, presented with polyuria, nocturnal bilateral foot spasms, and significant weight loss over the past three months. Laboratory results revealed a serum glucose of 822 mg/dL, HbA1c of 12.9%, positive urine ketones, and an anion gap metabolic acidosis. Importantly, he tested negative for autoantibodies, and his C-peptide level was low. These findings do not clearly categorize the patient into either type 1 or type 2 diabetes. However, a recently recognized form of diabetes, ketosis-prone type 2 diabetes mellitus, could explain the presentation. This subtype, which can present with diabetic ketoacidosis (DKA), is characterized by low C-peptide levels and negative autoantibodies, and typically involves elevated glucose (500-700 mg/dL), increased ketones, and high HbA1c levels. Notably, unlike type 1 diabetes, patients with ketosis-prone type 2 diabetes do not have beta-cell autoantibodies (^14,15^). The lack of a clear diabetes classification complicates the categorization of our patient’s condition, however the aforementioned clinical and laboratory features suggest he may fit the profile of ketosis-prone type 2 diabetes.

In conclusion, imatinib is widely used in the treatment of CML and gastrointestinal stromal tumors. In addition to its anti-proliferative properties, it has been shown to have a beneficial effect on glucose control in diabetic patients by lowering blood glucose, decreasing HbA1c levels, and reducing the need for anti-diabetes medications. However, we present a unique case of severe hyperglycemia and newly elevated HbA1c in a patient with no prior history of diabetes. This is noteworthy because numerous studies show that Imatinib is commonly known to reverse diabetes in patients who have developed it. This case underscores the importance of conducting additional research to assess the safety and effectiveness of imatinib on glucose metabolism, as well as the necessity for a more precise classification of diabetes.

## References

[1] Waller CF., Martens UM (2018). Small Molecules in Hematology.

[2] Markovits N, Kurnik D, Friedrich C, Gueta I, Halkin H, David S (2022). Effects of imatinib on glycemic and lipid profiles: a
retrospective cohort study. Leuk Lymphoma.

[3] Veneri D, Franchini M, Bonora E. (2005). Imatinib and Regression of Type 2 Diabetes. N Engl J Med.

[4] Lavelle K, Chamberlain C, German M, Anderson M, Nip A, Gitelman SE. (2024). The Role of Imatinib in Pediatric Type 1 Diabetes. JCEM Case Reports.

[5] Louvet C, Szot GL, Lang J, Lee MR, Martinier N, Bollag G (2008). Tyrosine kinase inhibitors reverse type 1 diabetes in nonobese
diabetic mice. Proc Natl Acad Sci USA.

[6] Jeon JY, Sparreboom A, Baker SD. (2017). Kinase Inhibitors: The Reality Behind the Success. Clin Pharmacol Ther.

[7] Claudiani S, Apperley JF. (2018). The argument for using imatinib in CML. Hematology.

[8] Cohen P, Cross D, Jänne PA. (2021). Kinase drug discovery 20 years after imatinib: progress and
future directions. Nat Rev Drug Discov.

[9] Gómez-Sámano MÁ, Baquerizo-Burgos JE, Coronel MFC, Wong-Campoverde BD, Villanueva-Martinez F, Molina-Botello D (2018). Effect of imatinib on plasma glucose concentration in subjects
with chronic myeloid leukemia and gastrointestinal stromal
tumor. BMC Endocr Disord.

[10] Salaroli A, Loglisci G, Serrao A, Alimena G, Breccia M. (2012). Fasting glucose level reduction induced by imatinib in chronic
myeloproliferative disease with TEL-PDGFRβ rearrangement and type 1
diabetes. Ann Hematol.

[11] Hägerkvist R, Sandler S, Mokhtari D, Welsh N. (2007). Amelioration of diabetes by imatinib mesylate (Gleevec): role of
beta-cell NF-kappaB activation and anti-apoptotic
preconditioning. FASEB J.

[12] Fitter S, Vandyke K, Gronthos S, Zannettino ACW. (2012). Suppression of PDGF-induced PI3 kinase activity by imatinib
promotes adipogenesis and adiponectin secretion. J Mol Endocrinol.

[13] Fountas A, Diamantopoulos LN, Tsatsoulis A. (2015). Tyrosine Kinase Inhibitors and Diabetes: A Novel Treatment
Paradigm?. Trends Endocrinol Metab.

[14] Smolenski S, George NM. (2019). Management of ketosis-prone type 2 diabetes
mellitus. J Am Assoc Nurse Pract.

[15] Umpierrez GE. (2006). Ketosis-Prone Type 2 Diabetes. Diabetes Care.

